# Consequences of the COVID-19 Lockdown in Germany: Effects of Changes in Daily Life on Musical Engagement and Functions of Music

**DOI:** 10.3390/ijerph181910463

**Published:** 2021-10-05

**Authors:** Natalie Alexa Roese, Julia Merrill

**Affiliations:** 1 Institute of Music, University of Kassel, Mönchebergstr. 1, 34125 Kassel, Germany; natalie.roese@mail.de; 2 Max Planck Institute for Empirical Aesthetics, Grüneburgweg 14, 60322 Frankfurt am Main, Germany

**Keywords:** music listening, pandemic, coronavirus, social distance, worries, killing time

## Abstract

The current study investigated how music has been used during the COVID-19 pandemic and how personal factors have affected music-listening behavior. During the shutdown in Spring 2020 in Germany, 539 participants took part in an online survey reporting on functions of music listening, attributes of listened music, and active engagement with music, retrospectively before and during the pandemic. Next to these implicit questions, participants were asked to describe the changes they explicitly noticed in handling music during COVID-19, their current worries, and their new everyday life during the pandemic as well as personality traits and stress reactivity. A logistic regression model was fitted, showing that people reduced their active engagement with music during the lockdown, and the function of killing time and overcoming loneliness became more important, reflecting the need for distraction and filling the silence. Before the lockdown, music was listened to for the function of motor synchronization and enhanced well-being, which reflects how people have lost both their musical and activity routines during the lockdown. The importance of in-person engagement with music in people’s lives became particularly evident in the connection between worries about further restrictions and the need for live music.

## 1. Introduction

The coronavirus disease 2019 (COVID-19) has brought new challenges to modern life. Through the influence of the media, without precedent, we have been able to follow the daily development and the impact of the virus on people’s lives. Every single person can probably tell in some way or another how the virus has affected their lives. With the current study, we investigated the role of music in these challenging times with a specific focus on retrospectively assessed changes from before and during the first lockdown in Germany. Hence, we took into account the particular situation in this country during the lockdown and related it to known music listening habits and functions of music in daily life.

### 1.1. The Situation in Germany at the Start of the Pandemic

When lockdown in Germany started on 13 March 2020, stores, restaurants, bars, and discos had to close. Parties and (sporting, music) events had to be cancelled, and schools and universities had to change to online lessons and homeschooling. Many people had to work from home and reduce their working hours, and some lost their jobs. The unemployment rate in Germany rose by 0.7% from March to April, and there were 10.1 million applications from employers to temporarily reduce employee working hours between March to April. Therefore, the number of people working fewer hours increased to an unprecedented level in Germany during this time [1].

From 13 March until 5 June 2020, people were only allowed to meet people from one other household. Between March and May, there were 181,482 infected people in Germany, and 8500 people had died of SARS-CoV-2 [2]. Through the media, the people in Germany were confronted with the possibility of an increase in the development of the COVID-19 pandemic comparable to other, strongly affected regions in the world. Based on the proportion of infected people and number of ICU stations, the German government justified the lockdown (and has ever since) to reduce the risk as much as possible of being in a position where the healthcare system has to make ethical judgements about whom to grant intensive care.

There is mixed evidence of the effect of consequences due to COVID-19 on the mental health of the German population. Entringer and Kröger (2020) [3] reported an increase in subjective loneliness during the first months of the restrictions in Germany, which was described as a discrepancy between desired and existing social relationships. Otherwise, the authors report that life satisfaction, emotional well-being, and symptoms of depression and anxiety were, interestingly, unchanged. People were even more satisfied with their health, which was probably because of the contrast to people who were infected with COVID-19 and suffered from health problems of SARS-CoV-2.

In a study by Bäuerle et al. (2020) [4], a high prevalence of generalized anxiety symptoms, depression symptoms, psychological distress, and COVID-19 related worries were seen from March until May, which shows that there was an increased mental health burden during the lockdown (although with a lower prevalence compared to China, which was also investigated). While in times before the pandemic, healthy people were shown to spend 28–55 min worrying [5,6], during the lockdown, people indicate on average spending 4.45 h per day thinking about COVID-19 and its effects [7]. The reported worries were more related to social than to personal aspects; that is, people were more concerned about social consequences than about getting infected or dealing with changes in their daily lives. Additionally, social consequences weighed more than the fear of economic consequences, and people indicated that anxiety due to the pandemic impacted their lives [7].

### 1.2. Music Use and Functions in Daily Life

That changes in daily life and music listening behavior go hand in hand has been shown in previous work. Music in general can take on an important role in everyday life, or at least as important as other domains, such as hobbies or food preferences [8]. In comparison to other leisure activities, music listening is the most preferred activity compared to sports, TV, books, movies, radio, and magazines or newspapers (e.g., people spend more money on music than on other activities; [9]). Music is often used simultaneously with other activities in daily routine [8,10,11,12,13,14], many of which have been missing during the COVID-19 pandemic [15].

Music in particular is known to be able to regulate mood and arousal [9,16], to cope with negative feelings [11,14,17], to express emotions [16], and to trigger memories and emotions [14,18,19]. Next to talking to friends, music is the second most important strategy for affect regulation [14]. These functions of music listening seem to come into play particularly when people want to change a negative mood or stress [11,14], because then, mood regulation is more important [20]. Certainly, music affects people differently, for example, depending on trait aspects such as personality, where people higher in openness and (slightly) extraversion are also higher in musical sophistication [21,22], but also depending on personal distress, where music can lead to a reduction of arousal and therefore, having a positive effect on fear, anxiety (e.g., [23] Daniel, 2016; [24] Knight and Rickard, 2001), and stress (e.g., [25] Hodges, 2010; [26] Kreutz et al., 2013).

Based on these primarily positive effects, it can be assumed that people use music to counteract the predominantly negative effects of the lockdown (see [3,4,7]). Music might be listened to because of its transformative power, that is to change cognitive, bodily, and self-conceptual states as well as one’s energy level [27,28].

### 1.3. Current Studies on Musical Behavior during the COVID-19 Pandemic

As shown, musical behavior is closely tied to habits and routines in everyday life. Fitting to this, a growing body of research in the past few months has given rise to the idea that music listening and making have changed together with changing habits and routines and adapted to the new way of living during the pandemic.

On the one hand, current studies have shown that music streaming volume decreased in several countries after the start of the lockdown [29], or more generally, by 12.5% after the WHO’s pandemic declaration [30]. The decline in music consumption was related to an increasing number of COVID-19 cases; in countries which recovered quickly, the consumption of music grew again [30].

On the other hand, Fink et al. (2021) [31] queried representative samples from six countries about musical behavior during the first lockdown (April–May 2020) and found that particularly the functions of music play an important role in socio-emotional coping during the lockdown as well as music selection behavior toward the so-called “coronamusic.” The most important functions of music listening during the lockdown were, after “interest in others’ coronamusic behavior,” “makes feel like having company,” and “reduces loneliness.” Some leisure activities ranked higher than music listening (which was ranked 6th), such as calling people, reading/watching news and movies/series, cleaning and cooking. Between 34% and 57% of the participants report about adapted musical behavior during the lockdown.

Mas-Herrero et al. (2020) [32] queried people from mainly three (Western) countries and found that music listening was the major coping strategy for regulating distress during the pandemic, and depression symptoms (The Depression Anxiety Stress Scale; DASS-21) decreased with the amount of music listening.

In another extensive study from eleven countries during the lockdown [33], music was found to be very efficient at attaining the goals (or functions) of enjoyment and maintaining a good mood, reducing loneliness, and creating a sense of togetherness (only socializing was higher, hobbies were equally good), releasing and venting negative emotions, connecting with oneself and detaching from the surroundings, and diversion from the crisis (note that entertainment media were equally efficient). Here, people scoring higher in the DASS-21 chose music more often, which was associated with nostalgia.

Investigating Spanish citizens in their musical behavior during the lockdown [34], another study observed a perceived increase in time spent on musical activities (making and listening) and how music was perceived to help coping for confinement: that is, to relax, escape, raise their mood, or keep them company. While these findings were based on descriptive statistics, inferential statistics were used to show that the employment situation had an impact on the perception of value of music, which was lowest for the retirees as compared to the other groups.

In light of the methods applied in the present study, it is of interest to note the methodological strategies used in these studies to investigate changes in musical behavior from before to during the lockdown. All four studies assessed changes by indirectly asking for perceived changes, e.g., “Since corona crisis measures were introduced, do you now listen to more or less music?” [31] or “How much time did you spend on listening to music during lockdown as compared to the time before the crisis? (much less–much more)” [33,34]. While Mas-Herrero et al. (2020) [32] used the same approach for the majority of the questions, one difference was seen with regard to a question on listening time, which was asked twice: once regarding before and once during the lockdown (note, comparisons were not made between these two points of measurement but only within).

Therefore, the changes between before and during the lockdown have been investigated rather indirectly with one question asking about perceived changes. Since a comparison between both time points is not possible (unless data were collected right before the lockdown), the only other possibility is to query the behavior from before the lockdown retrospectively and then compare the ratings to the same questions asked about the situation during the lockdown. This would allow for the comparison of the same questionnaires assessed for two different time points.

### 1.4. The Present Study

With the current study, we take an exploratory approach to examine music-listening behavior during the COVID-19 lockdown in Germany via an online survey. The primary goal was to compare the self-reported music listening behavior before and during the lockdown. Therefore, the active engagement with music (Goldsmiths Musical Sophistication Index, Gold-MSI; [35]), functions of music [10], and attributes of music [36] were assessed, once retrospectively before the pandemic and once during the lockdown.

First, changes in functions of music and in active engagement with music were to be expected due to changes in habits and routines during the lockdown. With the choice in assessments, a form of active engagement was investigated that goes beyond just time spent on music listening but includes aspects on seeking music-related information. The functions investigated in the present study covered a wide range including killing time and overcoming loneliness, mind wandering and emotional involvement, motor synchronization and enhanced well-being, and intellectual stimulation.

Second, because music with distinct attributes is often selected for its capacity to affect one’s mood, it was expected that changes in overall emotional states affect music selection criteria. The investigated attributes in the current study displayed three categories related to positive valence/joy, arousal/stimulative, and ‘depth’ (reflective, clever, emotional; see [36]).

Third, the observed changes in musical behavior were to be investigated depending on the personal situation. Therefore, aspects of changes in daily life and the worries related to the lockdown measures were collected. Items were based on daily topics that moved people in Germany at the time in April 2020 covered by the media and taken from statistical reports by the German government. Trait aspects were of interest that indicate how people deal with stress (prolonged stress reactivity and reactivity to work overload) [32,33], and personality dimensions previously reported to be related to musical behavior, that is openness and extraversion [21,22], and negative emotionality, which represents aspects of anxiety, depression, and emotional volatility [37].

To conclude, the present study covers aspects comparable to other studies, but with a major difference in the methodological approach: that is, the retrospective assessment of the behavior from before the lockdown, which allows for an implicit comparison of changes in musical behavior.

## 2. Materials and Methods

### 2.1. Ethics Statement

All experimental procedures were approved by the Ethics Council of the Max Planck Society and were undertaken with written informed consent of each participant.

### 2.2. Participants

Five hundred and thirty-nine participants (55.1% female, 44.3% male, 0.6% non-binary, mean age = 33.18, *SD* = 13.81 years) finished the online survey. The sample was a convenience sample with participants aged 18 years and older. Participants who did not finish the survey (*N* = 168) were not considered further. Most of the participants (44.5%) had as the highest degree a college diploma, while 39.1% had a high school diploma. Almost half of the sample were students (43.8%), 31.2% were employees, 14.5% were self-employed or similar, 4.5% were retired, and 2.2% were unemployed.

### 2.3. Procedure

Participants were recruited via social media (mainly Facebook), the website of the affiliated university, and with a newspaper article in the local press. The survey was created with LimeSurvey. Data were collected between 6 April and 15 May 2020, which overlaps with the (first) shut down in Germany. The survey lasted about 20 min. There was no monetary compensation for participation.

### 2.4. Questionnaires

Participants gave information on their demographics (age, gender, education, profession), the number of people in their household, living situation (living alone, with family, partner, in a shared apartment) and the date of the beginning of the lockdown in their area.

### 2.5. Perceived Changes in Music-Listening Behavior

A custom questionnaire was developed that included twelve items, which evaluated how the participants perceived the changes before the lockdown versus during the lockdown with regard to various aspects of music-listening behavior on a 5-point Likert scale from strongly disagree (1) to strongly agree (5). These aspects included changes arising from the restrictions in place and resulting changes in daily routines during the lockdown: that is, media usage, missing live events, social distancing, and situations and reasons for listening to music. The items and the content was similar to other current studies on music and COVID-19 [31,34], i.e., asking indirectly whether a behavior was higher or lower during the lockdown than before.

### 2.6. Active Musical Engagement

This and the following two questionnaires were presented in two blocks, firstly with regard to the situation before the lockdown and then during the lockdown. The factor on ‘active engagement’ from the Gold-MSI [35,38] comprises nine items, measuring free time spent on musical activities, writing and reading about music, income spent on music, keeping track of new music, and openness to unfamiliar music as well as visited music events. Items were evaluated on a 7-point Likert scale from strongly disagree (1) to strongly agree (7). That means that this factor is built on a broad concept of being engaged with music not only focusing on music listening but excluding music making. Note that the question on visited live events was only evaluated in the questionnaire regarding before the lockdown. This was followed by a list of musical styles, asking the participants to choose the styles they normally/currently listen to.

### 2.7. Attributes of Chosen Music

Items were chosen from the three factors of (ascribed) musical emotions and attributes of music from Fricke and Herzberg (2017) [36]. From the first factor, the attributes reflective, emotional, and clever were chosen (“depth”). From the second factor, the attributes fast, energetic, and voice were chosen (“stimulative”). From the third factor, the attributes uplifting, cheerful/happy, rhythmical, and relaxing were chosen (“joy”). The attributes were evaluated on a 5-point Likert scale from disagree (1) to agree (5).

### 2.8. Functions of Music Listening

From the original five factors in Greb et al. (2018) [10], four factors were of interest for the current study from which items were chosen that did not show cross-loadings on other factors in the original study. One item described if music is used for ‘intellectual stimulation’ (all others had cross-loadings). The factor ‘mind wandering/emotional involvement’ (six items) included items on music triggering imagination, emotions, and goosebumps, and creating a situation to understand, forget, or remember situations. The factor ‘motor synchronization/enhanced well-being’ (six items) consisted of how music triggers movement, enhances the mood, and enables listeners to feel fit, blow off steam, and sing along. The last factor ‘killing time/overcoming loneliness’ (three items) consisted of dealing with boredom and loneliness, and in connection with that, having music playing in the background. Since the factor ‘updating one’s musical knowledge’ showed overlap with the above Gold-MSI factor, it was left out. Items were evaluated on a 5-point Likert scale from does not apply (1) to fully applies (5).

### 2.9. Traits

After the music-related questionnaires, personality traits were assessed with the facets of open-mindedness, extraversion, and negative emotionality from the BFI-2 [39]. From the Perceived Stress Reactivity Scale [40], the factors ‘prolonged reactivity’ and ‘reactivity to work overload’ were selected.

### 2.10. Worries and Changes in Everyday Life

One custom questionnaire evaluated possible worries in relation to the impact of the lockdown (ten items), and another evaluated the changes in everyday life (14 items), which were evaluated on a 5-point Likert scale from does not apply (1) to fully applies (5). They were created based on daily topics that moved people in Germany at the time in April 2020 covered by the media and taken from statistical reports by the German Federal Statistics Office (www.destatis.de; accessed on 10 August 2021), and the platform www.statista.de (accessed on 10 August 2021).

Finally, participants were asked whether the impact of the lockdown had a pleasant effect on their work life (yes/no) and if they were working from home (yes/no/partly), if they were happy about that, and if they had more free time due to working from home (5-point Likert scale from does not apply (1) to fully applies (5)). Questions followed on supervised children, i.e., how many (0, 1, 2, 3, 4, 5, and more), and at which times (in the morning, the afternoon, the evening, the whole day), and if participants actually followed the instruction to stay at home if possible (yes/no).

### 2.11. Preprocessing and Factor Analyses

For active engagement, attributes, and functions, mean scores of the original factors were computed twice per person: once for the questionnaires describing the situation before and once during the lockdown. All analyses were performed using R Statistics 3.5.1.

The factor structure of the other questionnaires was determined with a factor analysis using oblimin rotation. Parallel analysis was used to determine the number of factors to keep in the factor analysis. Items with loadings < |0.3| were excluded (three items) as well as one factor that did not explain much of the variance and consisted of the two items on social contacts which only applied to a small percentage of the participants. After repeating the factor analysis without these items, factor scores were extracted, and latent variables were created to be used in the statistical models. Descriptive statistics, the details of the factor analysis, and the correlations of all latent variables can be found in the Supplementary Materials. The multicollinearity of each model was checked with variance inflation factors (VIF), the vif() function, accepting VIFs < 3.

### 2.12. Perceived Changes in Music-Listening Behavior

The first factor ‘music use’ combines items that describe the different listening situations, media use, and reasons for listening to music. The second factor ‘value of music’ comprises items about the importance of music in general and having more time to engage with music. The factor ‘live music’ contains the two items on missing live music and listening more to recorded music (Table 1).

### 2.13. Worries

The four factors describe uncertainties about the future, worry of subsistence, further restrictions, and of becoming infected with COVID-19 (Table 2).

### 2.14. Everyday Life

The two factors describe work-related changes (having more to do and the professional life becoming more stressful) as well as changes in private life (such as being alone, bored, or spending more time for oneself or the family) (Table 3).

### 2.15. Statistical Analysis

A logistic regression model was fitted predicting behavior from before (0) or during the lockdown (1) with the factors of active engagement, functions of music listening, and attributes of chosen music. For further analyses, a change score for each of these factors was created by subtracting the values from before the lockdown from the ones during the lockdown. Then, eight linear models were fitted with the factors of everyday life, worries, personality, and stress reactivity, predicting each of these change scores. The results are to be interpreted as changes in behavior from before to during the lockdown: In case of a positive estimate, the behavior is currently higher than normal. In case of a negative estimate, the behavior is currently lower than normal.

Similarly, three further linear models were fitted predicting the factors of perceived changes in musical behavior (music value, music use, live music) with the factors of everyday life, worries, personality, and stress reactivity.

## 3. Results

### 3.1. Descriptive Statistics

In different sections of the questionnaire, participants had to describe effects of the lockdown on their daily lives (job and living situation, changes in everyday life, worries) and on their music-listening behavior.

#### 3.1.1. Overall Living and Job Situation during the Lockdown

The vast majority of participants reported staying mainly at home (92.4%), and most did not have to take care of children (87.4%). Regarding living arrangements, 17.1% lived alone, 30.9% with their partner, 41.3% lived together with their family, 6.7% lived together with friends, and 3% lived in a flat-sharing community. Many participants perceived the impact of the lockdown on their work situation as unpleasant (73.7%), 64.6% were at least partly working from home during the lockdown, 75% reported being glad to have the opportunity to work from home, and 62% reported having at least partly more free time because of their job situation.

#### 3.1.2. Reported Changes in Everyday Life

Half of the participants are at least partly more often alone (52.9%) and have more time for themselves than before the lockdown (60.1%); 52.4% can at least partly not pursue their hobbies and 68.8% find the current restrictions somewhat decelerating. The family life and the professional life has at least partly become more stressful for 51% and 48.6% of the participants, respectively, and 51.9% report having more to do now than before the lockdown. Nonetheless, half of the participants report only slightly missing social contacts or having less contact with people who are important to them (51.9% and 56.6%, respectively; Table 4).

#### 3.1.3. Reported Worries during the Lockdown

Worries due to the COVID-19 pandemic are overall small to moderate with a median of 2 and an interquartile range (IQR) of 1–3 on the 5-point Likert scale (see Supplementary Materials). Most (89.6%) of the participants do not or only partly have existential fear. A similar percentage (83.3%) is seen for financial worries (“I am afraid of financial losses”). The fear of professional changes is low: 75.2% are not or only partly afraid of professional changes. Most of the participants do not or only partly worry about the personal future (77.4%) and about uncertainties about the future (72.7%). Worries about restrictions are rare in our sample with 80.7% (“I am afraid of further restrictions”) and 84.4% (“The restrictions of public life seem threatening”). Most (80.9%) of the participants are not or only partly afraid of falling ill themselves, but 82.9% are at least partly afraid that a relative or acquaintance will become infected with COVID-19, representing the most prominent worry (Table 5).

#### 3.1.4. Perceived Differences in Music-Listening Behavior

There are perceived changes concerning music-listening behavior during the lockdown: 44.4% perceived to at least partly listen to music in other situations and 36.9% for other reasons than before. The most extreme categories (“I listen to music in other situations”) were chosen by 26.5% (“does not apply”) and 5.8% (“fully applies”). Similar results were seen for reasons for music listening (“I listen to music for other reasons”). Only 2.8% indicate to agree; however, 31.4% disagree. Less than half (41.8%) indicate using other entertainment media to listen to music than before the lockdown and 54.8% indicate missing situations in which they typically listen to music.

Furthermore, there are perceived changes that describe the value of music during the lockdown: for 66.6% of the participants, music is more or at least partly more important during the lockdown. Most (75.9%) indicate that they are at least partly happy to have more time to listen to music; 24.3% totally agree and only 8.3% totally disagree. Half of the participants (53.5%) listen more often or at least partly more often to familiar music, but the most extreme category (“fully applies”) is only chosen by 5% in contrast to 19.9% (“does not apply”). More than one-third (36.9%) listen at least partly to music to compensate for missing daily activities. Here again, the most extreme category (“fully applies”) is only chosen by 5%. Most (82.6%) of the participants use entertainment media more often than before the lockdown. Different music is at least partly listened to by 30%.

The absence of live music seems to be a strong change in the participants’ lives: 68.6% miss live music at least partly during the times of confinement; 35.4% strongly agree. Half of the participants (53%) at least partly agree with the statements that they use records to compensate for missing live events (Table 6).

To summarize these findings, we see that between 40% and 50% of the participants report about changes in their everyday lives. Even though the participants in our sample do not suffer from major worries except for worrying that family members or acquaintances might get infected with COVID-19, the impact of the lockdown on daily lives is noticeable. Participants report about changes such as being more often alone or not being able to pursue their hobbies as well as professional changes such as working from home and having more to do than before the lockdown.

Changes in music behavior can be seen regarding media usage, now that live music has become astray, which is missed by many participants. In addition, participants show an adapted music-listening behavior, i.e., concerning situations in which they listen to music and reasons why they do. More than half of the participants indicate missing situations in which they usually listen to music. This rather reflects the consequences of changes in everyday life (routines and habits as well as getting used to a “new normal”) than worries that were caused by COVID-19.

### 3.2. Inferential Statistics

#### 3.2.1. Factors Predicting Changes in Musical Behavior before and during the Lockdown

The logistic regression models predicting music-related behavior before and during the lockdown showed a significant effect of active engagement (Gold-MSI) revealing that participants were less musically engaged during the lockdown (Odds Ratio = 0.85, *CI* = 0.75–0.95, *p =* 0.005, Tjur’s *R*^2^ = 0.034). Furthermore, during the lockdown, music was used more to kill time and to overcome loneliness (Odds Ratio = 1.25, *CI* = 1.08–1.45, *p* = 0.003), while before the lockdown, participants listened to music for motor synchronization and to enhance their well-being (Odds Ratio = 0.75, *CI* = 0.61–0.92, *p* = 0.006). Musical attributes did not predict changes in behavior.

To be comparable to other studies, it is of note that 30.6% of the participants listened to music for more than two hours every day during the lockdown, while only 23.6% of the participants listened to music for that long before the lockdown. Hence, music listening time alone slightly increased during the lockdown (see Supplementary Materials).

#### 3.2.2. Effects of Worries, Everyday Life, Personality, and Stress Reactivity on Listening Behavior

The fitted linear models predicting attributes of music showed that depth is positively predicted by the factors ‘private’ and open-mindedness and negatively by the factors ‘uncertainty’ and ‘work’ (Table 7). The models predicting changes in functions of music from before to during the lockdown showed a very low explained variance and could not be interpreted any further (*R*^2^ adjusted between −0.003 and 0.010, see Supplementary Materials). Active engagement is not predicted by any factors (*R*^2^ adjusted = −0.005, see Supplementary Materials). The models predicting perceived changes in musical behavior showed that extraversion predicts music use, ‘uncertainty’ predicts the value of music, and ‘restrictions’ and open-mindedness predict live music (Table 8). Stress reactivity did not become a significant predictor in any model.

## 4. Discussion

**Figure 1:**
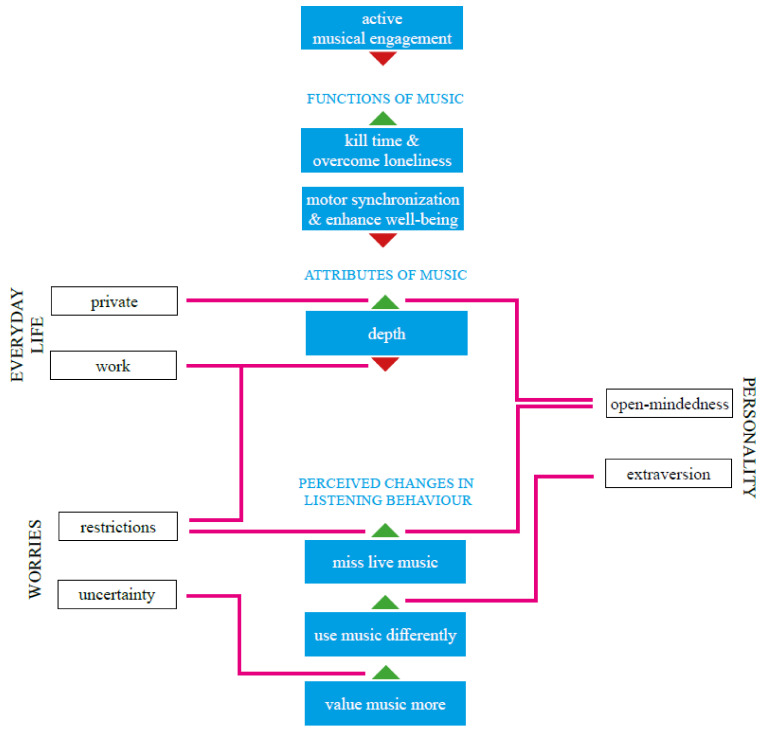
Changes in musical behavior during the lockdown and effects of worries, everyday life, and personality.

This study investigated music-listening behavior during the lockdown due to the COVID-19 pandemic from April to May 2020 in Germany. In the current study, we put the emphasis on the direct comparison between aspects of music-listening behavior (active musical engagement, functions of music listening, attributes of chosen music) from before and during the lockdown. The current study shows how music-listening behavior adapts to changed daily routines and habits as well as to the burdens of the lockdown. A summary of the results is depicted in Figure 1.

The figure shows factors significantly predicting changes in musical behavior from before to during the lockdown (top three boxes) and effects of worries, aspects of everyday life, and personality dimensions on music listening behavior (bottom boxes, pink lines). Green arrows show an increase during the lockdown, red arrows a decrease during the lockdown.

### 4.1. Decrease in Active Musical Engagement and Changes in Functions of Music Listening

Active engagement was investigated with a broad concept of being engaged with music, such as time spent on musical activities, including writing and reading about music, but excluding music making (Gold-MSI; [35,38]). We saw an overall decrease in active engagement with music during the lockdown compared to before.

If current behavioral studies report on changes in musical behavior before and during the lockdown, they have shown that the majority of participants listened to music as much or more than before restrictions [34] or show a perceived increase in musical activity [31,32], i.e., between 34% and 57% of the participants reported about adapted musical behavior during the lockdown. This is comparable to the current study, where participants report about changes in musical behavior related to different listening situations (30%) and reasons for music listening (37.6%), and missing situations in which they otherwise listen to music (54.8%). However, when comparing these results between the studies, the different assessments of musical activity need to be taken into account, as, for example, the pure listening time also slightly increased in the current study but not the overall engagement with music as measured with the Gold-MSI. By assessing active musical engagement with this standardized measure, it is investigated as a broad phenomenon and not reduced to music listening time. As no other study used this particular factor of an otherwise often cited assessment of musical sophistication, and the current study design differed from previous studies (retrospective assessment of behavior from before the lockdown), comparisons are not entirely valid.

However, the decrease in active engagement in music reflects other findings in relation to the lockdown such as a decrease in music streaming [29,30] and might be explained by the finding that people in Germany spent on average over four hours per day thinking about COVID-19 [7], indicating a permanent distraction that leaves little time and space for musical engagement.

Therefore, the reported changes in everyday life in the present study, such as missing the possibilities to pursue hobbies (52%) and changes in the working situation (64.6% working at least partly from home), have affected daily routines and habits, i.e., activities that have been shown to be tightly connected with music listening (e.g., [8,10,14]). Hence, these constraints have obviously affected music-listening routines, which need a new adjustment over time. Some adjustments could be seen in the current study, which comprise two important functions of music listening.

First, people listen to music more for the function of killing time and overcoming loneliness. Music being used to cope with negative feelings [9,16], for distraction, for filling the silence, and overcoming loneliness [9,10] are well-known functions of music listening, which were also found to be important in other recent investigations on the COVID-19 pandemic (e.g., [31,33]). Although our sample is not that strongly affected by worries concerning COVID-19, concern about changes in daily life as well as worrying that relatives and acquaintances will become infected show that participants had to deal with major constraints in their lives. Changes in private life concerning spatial distancing such as being bored (here, 52.4%) and often alone (52.9%) show moreover that people had to adapt to a “new normal”. Additionally, the current study shows that for 66.6% of the participants, music is more important, which fits with the results of Fink et al. (2021) [31] and Mas-Herrero (2020) [32], who show that music is used to deal with the consequences of the pandemic and may reflect the role of music serving as a socio-emotional and distress-regulating coping strategy during troubling times.

Second, in contrast to before the lockdown, music was listened to for the function of motor synchronization and enhanced well-being during the lockdown (i.e., how music triggers movement, enhances the mood, and enables listeners to feel fit, let off steam, and sing along). Even though it was previously shown that music, in comparison to other activities such as entertainment media, was found to be the most effective activity for three out of five well-being goals, that is enjoyment, venting negative emotions, and self-connection [33], the current results show how some of these goals have changed from before to during the lockdown. Furthermore, the function used in the current study connects well-being to certain situations in which music activates people (e.g., at parties and musical events, or at the gym). As about half of the current participants miss situations in which they usually listen to music and about a third indicate listening to music in different situations and for other reasons than before, this finding contributes to the assumption that people have lost their musical as well as their activity routines during the lockdown [15]. Findings by Fink et al. (2021) [31] also show that music-listening situations changed for 42% of the participants to the extent that private music listening sessions became more likely compared to social music listening events.

### 4.2. Changes in Musical Behavior Depending on Personal Situation

The current results show only a few significant effects of the personal situation on music-listening behavior. Interestingly, the attribute of depth in music was one of the few significantly predicted changes: Music with depth was chosen by people who reported about changes in private life and those higher in open-mindedness. Similarly, people higher in open-mindedness and those with higher fear of further restrictions also miss live music more than others. Previous research has shown that people higher in openness were linked to self-reported musical sophistication and are known for their higher engagement and interest in music and the arts [21,22]. Granot et al. (2021) [33] also found that because music is more important for people higher in openness, the music’s efficiency is also higher during the lockdown. Hence, these are the ones in the current study who miss the possibility of a higher engagement with music more strongly (such as live events), and listen to music with depth (reflective, emotional, clever music), reflecting their perceptiveness of emotion expression in music (e.g., [41], in the context of sad music).

On the other hand, people being worried by uncertainties about the future and reported changes in work life listened less to music with depth than before the lockdown. Being occupied with work or worries about the future has the effect that these people do not have the mental capacity to engage in a content-related confrontation with music (see [42,43]). Nonetheless, people who worry about the future value music more in general during the lockdown. Since private music listening provides one of the possibilities to engage with music during the lockdown, it can be used to convey some sort of normalcy and familiarity (e.g., half of the participants report listening to more familiar music during the lockdown). Particularly these people who fear an uncertain future possibly seek security or distraction by musical engagement without deeper confrontation (and therefore avoid music with depth).

Furthermore, people higher in extraversion, i.e., higher in sociability, energy level, and assertiveness [37], perceived using music differently during the lockdown. These results reflect the changes in daily life related to missing social (music listening) events and the resulting need to adapt music listening routines, as well as the need to compensate with different activities.

Overall, the small differences between differently affected groups can be explained with the current sample, which overall did not seem to be that worried by existential changes due to COVID-19 during the first lockdown in Germany. For example, while changes in the job situation are rated as unpleasant, people are still glad to have the possibility to work from home (probably to protect themselves from getting infected). Participants only partly suffer from more stress in daily family life compared to before the lockdown, which can be explained by most participants not having to take care of children and to being confronted with the balance between work and homeschooling. Furthermore, Giordano et al. (2020) [44] shows that for achieving well-being goals such as reducing tiredness, sadness, fear, and worries of clinical staff during the pandemic, the direct use of music (self-administration) is not effective compared to receptive music therapy. Hence, music listening without intervention might not be a useful strategy to deal with worries and changes in daily life in the current sample.

### 4.3. Limitations

The current study used a convenience sample typical for online surveys with well-educated participants and almost half being students. Only about 13% of the participants had to take care of children (note that in the representative sample of Fink et al. (2021) [31], about 31% lived with child(ren) and 11% had to do homeschooling), and most participants did not report having to face existential changes, which might partly explain why the worries were somewhat low in the current study. Nonetheless, changes in daily life were reported, which shows that the lockdown affected the people in the current study, presenting an important target to be investigated.

In general, using questionnaires to ask about music use and the time spent on music listening may lead to inaccurate responses, which is a limitation that has already been discussed in other COVID-19 and music-related studies (see, [12,34]), particularly when assessing musical behavior retrospectively as in the current study. Even though care was taken that the three questionnaires were assessed first on the retrospective view and then on the current view (and not the same one twice in a row), the responses might have been similar just because the participants remembered what they answered the first time. Still, it also means that participants had to become aware of their changes in behavior and directly compare them with before. Being aware of the strengths and limitations of this investigation, we have taken a unique approach to compare the behavior before and during the lockdown, which makes a valuable addition to other current investigations on this research topic.

The questionnaires on worries, everyday behavior, and perceived changes in music-listening behavior in the current study could not be validated beforehand due to the shortness of time (which is a typical problem in COVID-19 related studies, as discussed in Bäuerle et al. (2020) [4]). Care was taken that the items chosen covered daily topics present in the media and statistical reports of official channels in April 2020. Seeing strong overlap with other current approaches to music and COVID-19 [31,32,33] assured us that the selection was adequate.

## 5. Conclusions

Research has provided ample evidence on how various levels of music affect people’s everyday lives, but studies about musical engagement in exceptional and troubling times are rare. The study of the COVID-19 pandemic has given us a sad but unique opportunity to start filling in this gap: With the current study, we focused on changes from before and during the lockdown in Germany and on trying to find the connection between the restrictions applied and the changes in music-listening behavior. Overall, we see a decrease in musical engagement in the current sample, reflecting changes in daily routines and a lower capacity to engage with music due to the new challenges. Adjustments of music listening to the lockdown situation include music being used more to kill time and overcome loneliness and less for motor synchronization and enhanced well-being, reflecting a change in musical functions toward coping with loneliness and fewer possibilities to actively engage with music together with other people. In the same vein, many people report missing live music, particularly those worrying about further restrictions, showing the valuable effect of in-person engagement with music.

Finally, the results of the present study can give insights into how people use music during other exceptional circumstances such as death, illness, unemployment, or separation, in which people have to deal with similar worries and changes in daily life.

## Figures and Tables

**Table 1:** Factor solution of the items on perceived changes in music-listening behavior.

Item	Music Use	Value of Music	Live Music
Proportion variance explained	0.41	0.40	0.19
I listen to music in different situations than before.	0.98		
I use other media to listen to music than before.	0.87		
I listen to music for different reasons than before.	0.51		
I miss situations in everyday life, in which I would otherwise listen to music.	0.25		
Music is more important to me.		0.77	
I am happy to have time to listen to music.		0.62	
I listen to music I am familiar with more than before.		0.55	
I listen to music to compensate for missing everyday activities.		0.52	
I use media more often than before.		0.43	
I listen to different music than before.	0.30	0.35	
I miss listening to live music.			0.80
I use recordings more often because I cannot listen to live music.		0.35	0.51

For better readability, factor loadings < |0.3| are omitted. One item in the factor ‘music use’ with a loading < |0.3| was kept in order to prevent a loading of 1 on this factor.

**Table 2:** Factor solution of the items of the questionnaire on worries.

Item	Uncertainty	Subsistence	Restrictions	Sickness
Proportion variance explained	0.31	0.28	0.26	0.15
I am worried about my personal future.	0.82			
I am afraid of professional changes.	0.51			
I am afraid of uncertainties about the future.	0.44			
I am afraid of financial losses.		0.95		
I have existential fear.		0.49		
The restrictions of public life seem threatening to me.			0.80	
I am afraid of further restrictions.			0.66	
I am afraid of falling ill.				0.65
I am afraid that a relative or acquaintance will fall ill.				0.49

**Table 3:** Factor solution of the items on everyday life.

Item	Work	Private
Proportion variance explained	0.51	0.49
I have more to do than before the corona crisis.	0.84	
My professional life has become more stressful.	0.67	
I am more often alone.		0.44
I spend more time with people in my household.		0.43
I am bored more often.		0.43
I have more contact with friends and family.		0.35
I have more time for myself.		0.35
My everyday family life has become more stressful.		0.34
I find the current restrictions decelerating.		0.32
I can no longer pursue my hobbies.		0.30

**Table 4:** Reported changes in everyday life.

Item	Does Not Apply (in %)	Slightly Applies (in %)	Partially Applies (in %)	Strongly Applies (in %)	Fully Applies (in %)
I spend more time with people in my household.	15.8	46.9	21.7	8.0	7.6
My professional life has become more stressful.	41.9	9.5	9.6	15.6	23.4
I have more to do than before the corona crisis.	40.3	7.8	14.1	15	22.8
I have more time for myself.	13.4	26.5	29.7	15.6	14.8
I have less contact with people who are important to me.	3.2	56.6	24.3	10.2	5.8
I miss social contacts.	3.2	51.9	26.2	14.1	4.6
I find the current restrictions decelerating.	8.0	23.2	29.1	29.9	9.8
My everyday family life has become more stressful.	41.6	7.4	12.2	14.3	24.5
I have more contact with friends and family.	29.9	5.4	10.0	26.7	28.0
I am more often alone.	28.0	19.1	20.4	14.3	18.2
I can no longer pursue my hobbies.	15.6	32.1	20.8	18.6	13.0
I find the current reporting threatening.	21.2	5.8	13.5	30.2	29.3
I feel well informed.	4.5	22.6	40.6	24.9	7.4
I am bored more often.	38.0	9.5	15.4	16.3	20.8

**Table 5:** Reported worries during the lockdown.

Item	Does Not Apply (in %)	Slightly Applies (in %)	Partially Applies (in %)	Strongly Applies (in %)	Fully Applies (in %)
I am afraid of falling ill.	27.8	33.8	19.3	5.4	13.7
I am afraid that my relatives or aquaintances will fall ill.	4.1	13.0	17.4	28.8	36.7
I am afraid of professional changes.	31.9	26.2	17.1	8.9	16.0
I have existential fears.	52.3	28.0	9.3	3.7	6.7
I am afraid of financial losses.	41.2	29.5	12.6	6.5	10.2
I am afraid of supply shortages.	56.4	30.6	8.3	1.1	3.5
I am worried about my personal future.	32.3	24.7	20.4	9.6	13.0
I am afraid of further restrictions.	32.3	30.4	18.0	6.5	12.8
I am afraid of uncertainties about the future.	21.5	28.4	22.8	9.8	17.4
The restrictions of public life seem threatening.	37.5	31.7	15.2	5.2	10.4

**Table 6:** Perceived differences in music-listening behavior.

Item	Does Not Apply (in %)	Slightly Applies (in %)	Partially Applies (in %)	Strongly Applies (in %)	Fully Applies (in %)
Music is more important to me than before.	10.9	22.4	28.2	27.6	10.8
I am happy to have more time listening to music.	8.3	15.8	19.5	32.1	24.3
I miss live music.	17.6	13.7	11.5	21.7	35.4
I use records more often to compensate for missing live events.	23.9	23.0	16.5	22.4	14.1
I listen to different music than before.	38.6	31.4	17.4	10.0	2.6
I listen more often to familiar music than before.	19.9	26.7	24.9	23.6	5.0
I listen to music for other reasons than before.	31.4	31.7	16.1	18.0	2.8
I listen to music in other situations than before.	26.5	29.1	13.7	24.9	5.8
I miss situations in which I usually listen to music.	24.5	20.8	11.7	23.2	19.9
I listen to music to compensate for missing daily activities.	31.0	32.1	14.5	17.4	5.0
I use media more often than before.	7.4	10.0	10.0	38.6	34.0
I use other media to listen to music than before.	28.0	30.2	13.4	22.8	5.6

**Table 7:** Results of the linear models predicting changes in musical attributes.

	Joy			Depth			Stimulative		
Predictors	Estimates	*CI*	*p*	Estimates	*CI*	*p*	Estimates	*CI*	*p*
(Intercept)	−0.40	−0.90–0.10	0.121	−0.96	−1.50–−0.41	**0.001**	−0.13	−0.60–0.34	0.580
subsistence	0.03	−0.03–0.10	0.349	0.05	−0.03–0.12	0.221	0.04	−0.02–0.11	0.177
uncertainty	−0.05	−0.14–0.05	0.343	−0.14	−0.24–−0.03	**0.011**	−0.01	−0.10–0.08	0.770
restrictions	0.01	−0.06–0.08	0.707	0.03	−0.05–0.10	0.503	−0.02	−0.09–0.04	0.490
sickness	0.02	−0.07–0.10	0.709	0.00	−0.09–0.09	0.991	0.00	−0.08–0.08	0.996
work	−0.03	−0.10–0.04	0.419	−0.13	−0.21–−0.06	**0.001**	−0.02	−0.09–0.04	0.457
private	0.07	−0.01–0.15	0.079	0.14	0.06–0.23	**0.001**	0.02	−0.05–0.10	0.537
open-mindedness	0.02	−0.06–0.11	0.581	0.13	0.04–0.23	**0.006**	0.05	−0.03–0.13	0.259
extraversion	0.08	−0.01–0.17	0.073	−0.00	−0.10–0.09	0.955	0.02	−0.06–0.11	0.566
negativity	0.03	−0.07–0.12	0.598	0.05	−0.05–0.16	0.294	−0.02	−0.11–0.06	0.582
stress reactivity	0.02	−0.01–0.06	0.207	0.03	−0.01–0.07	0.129	−0.02	−0.06–0.01	0.176
stress workload	−0.01	−0.04–0.03	0.622	0.01	−0.03–0.05	0.528	0.01	−0.03–0.04	0.672
Observations	539			539			539		
*R*^2^/*R*^2^ adjusted	0.018/−0.003			0.052/0.033			0.017/−0.004		

Significant *p*-values are indicated in bold.

**Table 8:** Results of the linear models predicting perceived changes in musical behavior.

	Music Use			Value of Music			Live Music		
Predictors	Estimates	*CI*	*p*	Estimates	*CI*	*p*	Estimates	*CI*	*p*
(Intercept)	−0.81	−1.62–0.00	0.051	−0.59	−1.33–0.15	0.120	−1.07	−1.78–−0.36	**0.003**
subsistence	0.00	−0.11–0.11	0.967	−0.06	−0.16–0.04	0.212	0.02	−0.07–0.12	0.627
uncertainty	0.05	−0.10–0.21	0.515	0.16	0.02–0.30	**0.030**	0.00	−0.14–0.14	0.997
restrictions	0.09	−0.02–0.20	0.125	0.09	−0.01–0.20	0.078	0.16	0.06–0.26	**0.002**
sickness	0.02	−0.11–0.16	0.721	−0.07	−0.19–0.06	0.284	0.01	−0.11–0.13	0.901
work	0.06	−0.05–0.17	0.293	−0.08	−0.18–0.02	0.124	−0.04	−0.14–0.06	0.401
private	−0.03	−0.16–0.10	0.661	0.09	−0.02–0.21	0.118	0.10	−0.01–0.21	0.085
open-mindedness	−0.02	−0.16–0.12	0.736	0.05	−0.08–0.18	0.471	0.29	0.17–0.41	**<0.001**
extraversion	0.23	0.09–0.37	**0.001**	0.09	−0.04–0.21	0.194	0.07	−0.05–0.20	0.254
negativity	0.07	−0.09–0.22	0.395	0.06	−0.08–0.20	0.366	0.00	−0.13–0.14	0.966
stress reactivity	0.00	−0.06–0.06	0.950	0.02	−0.04–0.08	0.459	−0.03	−0.09–0.02	0.246
stress workload	−0.02	−0.07–0.04	0.566	−0.03	−0.08–0.02	0.208	−0.04	−0.09–0.01	0.132
Observations	539			539			539		
*R*^2^/*R*^2^ adjusted	0.038/0.018			0.044/0.024			0.085/0.066		

Significant *p*-values are indicated in bold.

## Data Availability

The data are included as Supplementary Materials.

## Supplementary Materials

The following are available online at https://www.mdpi.com/article/10.3390/ijerph181910463/s1, Supporting Information, Data, Questionnaire.
