# Supplementary material for: Consequences of the COVID-19 Lockdown in Germany: Effects of Changes in Daily Life on Musical Engagement and Functions of Music
Source: Int J Environ Res Public Health. 2021 Oct 5;18(19):10463. doi: 10.3390/ijerph181910463 (PMC8507817; doi:10.3390/ijerph181910463)
Supplement: Supplementary file 1 [file ijerph-18-10463-s001.zip › ijerph-1357417-supplementary.pdf]

Musikhörverhalten in der Covid-19-Krise

Mit dieser Studie untersuchen wir das Musikhörverhalten während der Covid-19-Krise im Vergleich zum bisherigen Hörverhalten. Zuerst beantworten Sie bitte Fragen zu Ihrer allgemeinen Einstellung zu Musik und inwiefern sich Ihre Einstellung aktuell während der Covid-19-Krise ändert. Des Weiteren werden Ihnen Fragen zu Ihrer Persönlichkeit und Ihrem Umgang mit Stress im Alltag gestellt. Abschließend folgen allgemeine Fragen zu Ihrem Umgang mit der Covid-19-Krise und Ihrer Situation.

Kontakt für mögliche Nachfragen: uk024755@student.uni-kassel.de

UNI KASSEL
VERSITÄT

In dieser Umfrage sind 40 Fragen enthalten.

Welchem Geschlecht fühlen Sie sich zugehörig? *

Bitte wählen Sie eine der folgenden Antworten:
Bitte wählen Sie nur eine der folgenden Antworten aus:

- ◯ weiblich
- ◯ männlich
- ◯ divers

Wie alt sind Sie? *

In dieses Feld dürfen nur Zahlen eingegeben werden.
Bitte geben Sie Ihre Antwort hier ein:

Welchen höchsten Bildungsabschluss haben Sie? *

❗ Bitte wählen Sie eine der folgenden Antworten:
Bitte wählen Sie nur eine der folgenden Antworten aus:

- ◯ Ich bin derzeit Schüler/in, besuche eine allgemeinbildende Vollzeitschule
- ◯ kein Abschluss
- ◯ Hauptschulabschluss
- ◯ Mittlere Reife
- ◯ Fachhochschulreife
- ◯ Allgemeine Hochschulreife/ Abitur
- ◯ abgeschlossenes Studium
- ◯ Promotion
- ◯ sonstiges

Welcher Tätigkeit gehen Sie zur Zeit nach? *

❗ Bitte wählen Sie eine der folgenden Antworten:
Bitte wählen Sie nur eine der folgenden Antworten aus:

- ◯ Schüler/in
- ◯ Auszubildende/r
- ◯ Student/in
- ◯ Arbeiter/in oder Handwerker/in
- ◯ Angestellte/r
- ◯ leitende/r Angestellte/r
- ◯ selbstständig
- ◯ verbeamtet
- ◯ Rentner/in
- ◯ arbeitslos
- ◯ sonstiges

Wieviele Personen leben in dem Haushalt, in dem Sie sich während der Covid-19-Krise hauptsächlich aufhalten? *

❗ In dieses Feld dürfen nur Zahlen eingegeben werden.
Bitte geben Sie Ihre Antwort hier ein:

In welcher Wohnform leben Sie zur Zeit der Covid-19-Krise hauptsächlich? *

❗ Bitte wählen Sie eine der folgenden Antworten:
Bitte wählen Sie nur eine der folgenden Antworten aus:

- ◯ alleine
- ◯ mit Familie
- ◯ mit Partner/in
- ◯ in einer WG mit Freunden
- ◯ in einer "Zweck-WG"
- ◯ Anderes

Mit den folgenden Aussagen beschreiben Sie ihren aktuellen Umgang mit Musik seit Beginn der Ausgangsbeschränkungen in Ihrer Region.

Wann begannen die Ausgangsbeschränkungen bei Ihnen? *

❗ Bitte wählen Sie eine der folgenden Antworten:
Bitte wählen Sie nur eine der folgenden Antworten aus:

- ◯ am 23.03.2020
- ◯ am 16.03.2020
- ◯ vor dem 16.03.2020
- ◯ Sonstiges ______

Seit Beginn der Ausgangsbeschränkungen ... *

Bitte wählen Sie die zutreffende Antwort für jeden Punkt aus:

	stimme überhaupt nicht zu	stimme eher nicht zu	teils, teils	stimme eher zu	stimme voll und ganz zu
**...ist mir Musik wichtiger.**	○	○	○	○	○
**...freue ich mich, Zeit zum Musikhören zu haben.**	○	○	○	○	○
**...fehlt es mir, Live-Musik zu hören.**	○	○	○	○	○
**...greife ich öfter auf Aufnahme zurück, da ich keine Live-Musik hören kann.**	○	○	○	○	○
**... höre ich andere Musik als vorher.**	○	○	○	○	○
**...höre ich häufiger mir bekannte Musik als vorher.**	○	○	○	○	○
**...höre ich aus anderen Gründen Musik als vorher.**	○	○	○	○	○
**...höre ich in anderen Situationen Musik als vorher.**	○	○	○	○	○
**...fehlen mir Situationen im Alltag, in denen ich sonst Musik hören würde (Weg zur Arbeit, Sport,...).**	○	○	○	○	○
**...höre ich Musik zur Kompensation fehlender Alltagsbeschäftigungen.**	○	○	○	○	○
**...nutze ich Medien häufiger als vorher.**	○	○	○	○	○
**...nutze ich andere Medien, um Musik zu hören als vorher.**	○	○	○	○	○

Wie gut stimmen Sie den Aussagen zu, mit denen Sie Ihr **normales Verhalten (also vor der Covid-19-Krise)** beschreiben? *

Bitte wählen Sie die zutreffende Antwort für jeden Punkt aus:

	Stimme ganz und gar nicht zu	Stimme nicht zu	Stimme eher nicht zu	Weder noch	Stimme eher zu	Stimme zu	Stimme voll und ganz zu
**Ich beschäftige mich normalerweise in meiner Freizeit viel mit musikbezogenen Aktivitäten.**	○	○	○	○	○	○	○
**Ich schreibe normalerweise gerne über Musik, z.B. in Internetblogs und Foren.**	○	○	○	○	○	○	○
**Ich bin normalerweise fasziniert von Musikstilen, mit denen ich nicht vertraut bin und möchte mehr darüber erfahren.**	○	○	○	○	○	○	○
**Ich lese und suche normalerweise oft im Internet nach Dingen, die mit Musik zu tun haben.**	○	○	○	○	○	○	○
**Ich gebe normalerweise nicht viel Geld meines verfügbaren Einkommens für Musik aus.**	○	○	○	○	○	○	○
**Musik ist normalerweise eine Art von Sucht- ohne sie könnte ich nicht leben.**	○	○	○	○	○	○	○
**Ich halte mich normalerweise auf dem Laufenden was neue Musik angeht (z.B. neue Künstler oder Aufnahmen).**	○	○	○	○	○	○	○

Ich habe ____ Live-Events als Zuschauer innerhalb der letzten 12 Monate besucht. *

Bitte wählen Sie eine der folgenden Antworten:
Bitte wählen Sie nur eine der folgenden Antworten aus:

- ◯ 0
- ◯ 1
- ◯ 2
- ◯ 3
- ◯ 4-6
- ◯ 7-10
- ◯ 11 oder mehr

Ich höre **normalerweise** jeden Tag aufmerksam Musik für _____ *

Bitte wählen Sie eine der folgenden Antworten:
Bitte wählen Sie nur eine der folgenden Antworten aus:

- ◯ 0-15 Minuten
- ◯ 15-30 Minuten
- ◯ 30-60 Minuten
- ◯ 60-90 Minuten
- ◯ 2 Stunden
- ◯ 2-3 Stunden
- ◯ 4 Stunden oder mehr

Welche Musikrichtungen hören Sie **normalerweise** hauptsächlich? *

Bitte wählen Sie die zutreffenden Antworten aus:
Bitte wählen Sie alle zutreffenden Antworten aus:

- [ ] Hip-Hop/Rap
- [ ] Pop
- [ ] Jazz
- [ ] Klassik
- [ ] Rock
- [ ] Metal
- [ ] R&B
- [ ] Soul
- [ ] Schlager
- [ ] Elektro
- [ ] K-Pop
- [ ] Deutschrap
- [ ] Mainstream
- [ ] House
- [ ] Dubstep
- [ ] Reggae
- [ ] Neue Musik
- [ ] Salsa/Latin
- [ ] Sonstiges: ________

Die Musik, welche ich **normalerweise** höre, ist ... *

Bitte wählen Sie die zutreffende Antwort für jeden Punkt aus:

	stimme nicht zu	stimme eher nicht zu	teils, teils	stimme eher zu	stimme zu
**entspannend**	◯	◯	◯	◯	◯
**gefühlsbetont**	◯	◯	◯	◯	◯
**energievoll**	◯	◯	◯	◯	◯
**aufmunternd**	◯	◯	◯	◯	◯
**nachdenklich**	◯	◯	◯	◯	◯
**düster**	◯	◯	◯	◯	◯
**fröhlich**	◯	◯	◯	◯	◯
**geistreich**	◯	◯	◯	◯	◯
**schnell**	◯	◯	◯	◯	◯
**rhythmisch**	◯	◯	◯	◯	◯
**mit Gesang**	◯	◯	◯	◯	◯

Ich höre **normalerweise** Musik, weil... *

Bitte wählen Sie die zutreffende Antwort für jeden Punkt aus:

	trifft nicht zu	trifft eher nicht zu	teils, teils	trifft eher zu	trifft zu
**sie eine intellektuelle Stimulation für mich ist.**	○	○	○	○	○
**sie mich an bestimmte Phasen meines Lebens bzw. an vergangene Ereignisse erinnert.**	○	○	○	○	○
**ich darin meine Gefühle und Stimmungen wiederfinde.**	○	○	○	○	○
**mir dann tolle Bilder oder Geschichten in den Kopf kommen.**	○	○	○	○	○
**ich dann Gänsehaut bekomme.**	○	○	○	○	○
**ich dann die Welt um mich herum vergessen kann.**	○	○	○	○	○
**ich mich dann fitter fühle.**	○	○	○	○	○
**ich mich dazu bewegen kann.**	○	○	○	○	○
**ich dadurch Dampf ablassen kann.**	○	○	○	○	○
**ich dabei mitsingen oder mitsummen kann.**	○	○	○	○	○
**sie meinen Stress reduziert.**	○	○	○	○	○
**ich damit Zeit totschlagen kann.**	○	○	○	○	○
**ich sie im Hintergrund brauche, während ich etwas anderes tue.**	○	○	○	○	○
**ich mich dann weniger einsam fühle.**	○	○	○	○	○

Nun geben Sie bitte an, inwiefern die folgenden Aussagen **im Moment (also während der Covid-19-Krise)** auf Sie zutreffen? *

Bitte wählen Sie die zutreffende Antwort für jeden Punkt aus:

	Stimme ganz und gar nicht zu	Stimme nicht zu	Stimme eher nicht zu	Weder noch	Stimme eher zu	Stimme zu	Stimme voll und ganz zu
**Ich beschäftige mich aktuell in meiner Freizeit viel mit musikbezogenen Aktivitäten.**	◯	◯	◯	◯	◯	◯	◯
**Ich schreibe aktuell gerne über Musik.**	◯	◯	◯	◯	◯	◯	◯
**Ich bin aktuell fasziniert von Musikstilen, mit denen ich nicht vertraut bin und möchte mehr darüber erfahren.**	◯	◯	◯	◯	◯	◯	◯
**Ich lese und suche aktuell oft im Internet nach Dingen, die mit Musik zu tun haben.**	◯	◯	◯	◯	◯	◯	◯
**Ich gebe aktuell nicht viel Geld meines verfügbaren Einkommens für Musik aus.**	◯	◯	◯	◯	◯	◯	◯
**Musik ist aktuell für mich eine Art von Sucht - ohne sie könnte ich nicht Leben.**	◯	◯	◯	◯	◯	◯	◯
**Ich halte mich aktuell auf dem Laufenden was neue Musik angeht (z.B. neue Künstler oder Aufnahmen)**	◯	◯	◯	◯	◯	◯	◯

Ich höre **aktuell** jeden Tag aufmerksam Musik für _____. *

Bitte wählen Sie eine der folgenden Antworten:
Bitte wählen Sie nur eine der folgenden Antworten aus:

- 0-15 Minuten
- 15-30 Minuten
- 30-60 Minuten
- 60-90 Minuten
- 2 Stunden
- 2-3 Stunden
- 4 Stunden oder mehr

Welche Musikrichtungen hören Sie **aktuell** hauptsächlich? *

Bitte wählen Sie die zutreffenden Antworten aus:
Bitte wählen Sie alle zutreffenden Antworten aus:

- Hip-Hop/Rap
- Pop
- Jazz
- Klassik
- Rock
- Metal
- R&B
- Soul
- Schlager
- Elektro
- K-Pop
- Deutschrap
- Mainstream
- House
- Dubstep
- Reggae
- Neue Musik
- Salsa/Latin
- Sonstiges:

Die Musik, welche ich **aktuell** höre, ist ... *

Bitte wählen Sie die zutreffende Antwort für jeden Punkt aus:

	stimme nicht zu	stimme eher nicht zu	teils, teils	stimme eher zu	stimme zu
**entspannend**	◯	◯	◯	◯	◯
**gefühlsbetont**	◯	◯	◯	◯	◯
**energievoll**	◯	◯	◯	◯	◯
**aufmunternd**	◯	◯	◯	◯	◯
**nachdenklich**	◯	◯	◯	◯	◯
**düster**	◯	◯	◯	◯	◯
**fröhlich**	◯	◯	◯	◯	◯
**geistreich**	◯	◯	◯	◯	◯
**schnell**	◯	◯	◯	◯	◯
**rhythmisch**	◯	◯	◯	◯	◯
**mit Gesang**	◯	◯	◯	◯	◯

Ich höre **aktuell** Musik, weil... *

Bitte wählen Sie die zutreffende Antwort für jeden Punkt aus:

	trifft nicht zu	trifft eher nicht zu	teils, teils	trifft eher zu	trifft zu
**sie eine intellektuelle Stimulation für mich ist.**	○	○	○	○	○
**sie mich an bestimmte Phasen meines Lebens bzw. an vergangene Ereignisse erinnert.**	○	○	○	○	○
**ich darin meine Gefühle und Stimmungen wiederfinde.**	○	○	○	○	○
**mir dann tolle Bilder oder Geschichten in den Kopf kommen.**	○	○	○	○	○
**ich dann Gänsehaut bekomme.**	○	○	○	○	○
**ich dadurch die Welt besser verstehen kann.**	○	○	○	○	○
**ich dann die Welt um mich herum vergessen kann.**	○	○	○	○	○
**ich mich dann fitter fühle.**	○	○	○	○	○
**ich mich dazu bewegen kann.**	○	○	○	○	○
**sie meine Stimmung verbessern kann.**	○	○	○	○	○
**ich dadurch Dampf ablassen kann.**	○	○	○	○	○
**ich dabei mitsingen oder mitsummen kann.**	○	○	○	○	○
**sie meinen Stress reduziert.**	○	○	○	○	○
**ich damit die Zeit totschlagen kann.**	○	○	○	○	○
**ich sie im Hintergrund brauche, während ich etwas anderes tue.**	○	○	○	○	○
**ich mich dann weniger einsam fühle.**	○	○	○	○	○

Nachstehend finden sie eine Reihe von Eigenschaften, die auf Sie zutreffen können. Würden Sie über sich z.B. sagen, dass sie gerne Zeit mit anderen Menschen verbringen? Bitte geben Sie für jede der folgenden Aussagen an, inwieweit Sie zustimmen. *

Bitte wählen Sie die zutreffende Antwort für jeden Punkt aus:

	stimme überhaupt nicht zu	stimme eher nicht zu	teils,teils	stimme eher zu	stimme voll und ganz zu
**Ich gehe aus mir heraus, bin gesellig.**	○	○	○	○	○
**Ich bleibe auch in stressigen Situationen gelassen.**	○	○	○	○	○
**Ich bin nicht sonderlich kunstinteressiert.**	○	○	○	○	○
**Ich bin durchsetzungsfähig, energisch.**	○	○	○	○	○
**Ich bleibe auch bei Rückschlägen zuversichtlich.**	○	○	○	○	○
**Ich bin vielseitig interessiert.**	○	○	○	○	○
**Ich schäume selten vor Begeisterung über.**	○	○	○	○	○
**Ich kann launisch sein, habe schwankende Stimmungen.**	○	○	○	○	○
**Ich bin erfinderisch, mir fallen raffinierte Lösungen ein.**	○	○	○	○	○
**Ich bin eher ruhig.**	○	○	○	○	○
**Ich reagiere leicht angespannt.**	○	○	○	○	○
**Ich kann mich für Kunst, Musik und Literatur begeistern.**	○	○	○	○	○
**Ich neige dazu, die Führung zu übernehmen.**	○	○	○	○	○
**Ich bin selbstsicher, mit mir zufrieden.**	○	○	○	○	○
**Ich meide philosophische Diskussionen.**	○	○	○	○	○
**Ich bin weniger aktiv und unternehmungslustig als andere.**	○	○	○	○	○

	stimme überhaupt nicht zu	stimme eher nicht zu	teils,teils	stimme eher zu	stimme voll und ganz zu
**Ich bin ausgeglichen, nicht leicht aus der Ruhe zu bringen.**	◯	◯	◯	◯	◯
**Ich bin nicht besonders einfallsreich.**	◯	◯	◯	◯	◯
**Ich bin eher schüchtern.**	◯	◯	◯	◯	◯
**Ich mache mir oft Sorgen.**	◯	◯	◯	◯	◯
**Ich weiß Kunst und Schönheit zu schätzen.**	◯	◯	◯	◯	◯
**Mir fällt es schwer, andere zu beeinflussen.**	◯	◯	◯	◯	◯
**Ich fühle mich oft bedrückt, freudlos.**	◯	◯	◯	◯	◯
**Es macht mir Spaß, gründlich über komplexe Dinge nachzudenken und sie zu verstehen.**	◯	◯	◯	◯	◯
**Ich bin voller Energie und Tatendrang.**	◯	◯	◯	◯	◯
**Ich habe meine Gefühle unter Kontrolle, werde selten wütend.**	◯	◯	◯	◯	◯
**Ich bin nicht sonderlich fantasievoll.**	◯	◯	◯	◯	◯
**Ich bin gesprächig.**	◯	◯	◯	◯	◯

Dieser Test fragt nach Ihren typischen Reaktionen auf Situationen, die Sie bestimmt schon erlebt haben. Es werden jeweils drei Antworten vorgeschlagen. Bitte kreuzen Sie die Reaktion an, die Sie normalerweise bei sich beobachten. Wenn Sie unsicher sind, entscheiden Sie sich für die Antwort, die für Sie am ehesten zutrifft. Lassen Sie bitte keine Frage aus, auch wenn es Ihnen manchmal schwer fällt, sich zu entscheiden.

Wenn sich Aufgaben so häufen, dass sie kaum zu schaffen sind, ... *

❗ Bitte wählen Sie eine der folgenden Antworten:
Bitte wählen Sie nur eine der folgenden Antworten aus:

◯ lasse ich mich im Allgemeinen nicht aus dem Gleichgewicht bringen.

◯ werde ich meist etwas unruhig.

◯ werde ich gewöhnlich ziemlich nervös.

Wenn ich nach einem anstrengenden Arbeitstag ausspannen möchte, ... *

❗ Bitte wählen Sie eine der folgenden Antworten:
Bitte wählen Sie nur eine der folgenden Antworten aus:

- ◯ fällt mir das meist ziemlich schwer.
- ◯ gelingt mir das meist einigermaßen.
- ◯ habe ich damit im Allgemeinen keine Probleme.

Wenn ich für meine Arbeit wenig Zeit habe, ... *

❗ Bitte wählen Sie eine der folgenden Antworten:
Bitte wählen Sie nur eine der folgenden Antworten aus:

- ◯ bleibe ich meist ruhig.
- ◯ werde ich meist unruhig.
- ◯ werde ich meist ziemlich hektisch.

Wenn ich nach anstrengender Arbeit Freizeit habe, ... *

❗ Bitte wählen Sie eine der folgenden Antworten:
Bitte wählen Sie nur eine der folgenden Antworten aus:

- ◯ fällt es mir oft schwer, abzuschalten und mich zu entspannen.
- ◯ braucht es meist einige Zeit, bis ich richtig abschalten kann.
- ◯ kann ich meist gut abschalten und die Probleme des Tages vergessen.

Wenn etwas nicht so läuft, wie ich es mir vorgestellt habe, ... *

❗ Bitte wählen Sie eine der folgenden Antworten:
Bitte wählen Sie nur eine der folgenden Antworten aus:

- ◯ bleibe ich meist gelassen.
- ◯ werde ich oft ungeduldig.
- ◯ könnte ich meist aus der Haut fahren.

Wenn ich viele Aufgaben auf einmal zu erledigen habe, ... *

Bitte wählen Sie eine der folgenden Antworten:
Bitte wählen Sie nur eine der folgenden Antworten aus:

- ◯ bleibe ich im Allgemeinen gelassen und mache eines nach dem anderen.
- ◯ werde ich meist ungeduldig, mir geht alles nicht schnell genug.
- ◯ machen mich meist schon kleine Störungen nervös.

Wenn ich unter Stress stehe, ... *

Bitte wählen Sie eine der folgenden Antworten:
Bitte wählen Sie nur eine der folgenden Antworten aus:

- ◯ kann ich die verbleibende Freizeit meist gar nicht mehr genießen.
- ◯ kann ich die verbleibende Freizeit meist nicht mehr richtig genießen.
- ◯ kann ich die verbleibende Freizeit meist trotzdem genießen.

Wenn sich Aufgaben so häufen, dass sie kaum zu bewältigen sind, ... *

Bitte wählen Sie eine der folgenden Antworten:
Bitte wählen Sie nur eine der folgenden Antworten aus:

- ◯ ist mein Schlaf wie immer.
- ◯ schlafe ich unruhiger als sonst.
- ◯ ist mein Schlaf deutlich schlechter.

Wenn ich sehr viele Verpflichtungen/Aufgaben zu erfüllen habe, ... *

Bitte wählen Sie eine der folgenden Antworten:
Bitte wählen Sie nur eine der folgenden Antworten aus:

- ◯ bleibe ich im Allgemeinen gelassen.
- ◯ werde ich meist ziemlich ungeduldig.
- ◯ werde ich oft richtig hektisch.

Wenn ich nach einem anstrengenden Arbeitstag ausspannen möchte, ... *

❶ Bitte wählen Sie eine der folgenden Antworten:
Bitte wählen Sie nur eine der folgenden Antworten aus:

- ◯ fällt mir das meist ziemlich schwer.
- ◯ gelingt mir das meist einigermaßen.
- ◯ habe ich damit im Allgemeinen keine Probleme.

Wie fühlten Sie sich in den letzten Tagen? *

Bitte wählen Sie die zutreffende Antwort für jeden Punkt aus:

	ganz wenig/gar nicht	ein bisschen	einigermaßen	erheblich	äußerst
**zufrieden**	◯	◯	◯	◯	◯
**energiegeladen**	◯	◯	◯	◯	◯
**gestresst**	◯	◯	◯	◯	◯
**müde**	◯	◯	◯	◯	◯
**friedlich**	◯	◯	◯	◯	◯
**unglücklich**	◯	◯	◯	◯	◯
**lustlos**	◯	◯	◯	◯	◯
**ruhig**	◯	◯	◯	◯	◯
**begeistert**	◯	◯	◯	◯	◯
**besorgt**	◯	◯	◯	◯	◯

Haben Sie Angst vor den möglichen Auswirkungen der Covid-19-Pandemie? *

Bitte wählen Sie die zutreffende Antwort für jeden Punkt aus:

	trifft nicht zu	trifft eher nicht zu	teils, teils	trifft eher zu	trifft zu
**Ich habe Angst zu erkranken.**	○	○	○	○	○
**Ich habe Angst, dass ein Angehöriger oder Bekannter erkrankt.**	○	○	○	○	○
**Ich habe Angst vor beruflichen Veränderungen.**	○	○	○	○	○
**Ich habe existenzielle Ängste.**	○	○	○	○	○
**Ich habe Angst vor finanziellen Verlusten.**	○	○	○	○	○
**Ich habe Angst vor Versorgungsengpässen.**	○	○	○	○	○
**Ich mache mir Sorgen um meine persönliche Zukunft.**	○	○	○	○	○
**Ich habe Angst vor weiteren Einschränkungen.**	○	○	○	○	○
**Die Unsicherheiten in Bezug auf die Zukunft machen mir Angst.**	○	○	○	○	○
**Die Einschränkungen des öffentlichen Lebens wirken auf mich bedrohlich.**	○	○	○	○	○

Wie hat sich Ihr Alltag während der Covid-19-Krise verändert? *

Bitte wählen Sie die zutreffende Antwort für jeden Punkt aus:

	trifft nicht zu	trifft eher nicht zu	teils, teils	trifft eher zu	trifft zu
**Ich verbringe mehr Zeit mit Personen in meinem Haushalt.**	◯	◯	◯	◯	◯
**Mein beruflicher Alltag ist stressiger geworden.**	◯	◯	◯	◯	◯
**Ich habe mehr zu tun als vor der Corona-Krise.**	◯	◯	◯	◯	◯
**Ich habe mehr Zeit für mich selbst.**	◯	◯	◯	◯	◯
**Ich habe weniger Kontakt zu Menschen, die mir wichtig sind.**	◯	◯	◯	◯	◯
**Ich vermisse soziale Kontakte.**	◯	◯	◯	◯	◯
**Ich empfinde die derzeitigen Einschränkungen entschleunigend.**	◯	◯	◯	◯	◯
**Mein Familienalltag ist stressiger/anstrengender geworden.**	◯	◯	◯	◯	◯
**Ich habe mehr Kontakt mit Freunden und Familie**	◯	◯	◯	◯	◯
**Ich bin häufiger allein.**	◯	◯	◯	◯	◯
**Ich kann meinen Hobbies nicht mehr nachgehen.**	◯	◯	◯	◯	◯
**Ich empfinde die derzeitige Berichterstattung bedrohlich.**	◯	◯	◯	◯	◯
**Ich fühle mich gut informiert.**	◯	◯	◯	◯	◯
**Mir ist häufiger langweilig.**	◯	◯	◯	◯	◯

Empfinden Sie die Auswirkungen der Covid-19-Krise auf Ihren Job als angenehm? *

❗ Bitte wählen Sie eine der folgenden Antworten:
Bitte wählen Sie nur eine der folgenden Antworten aus:

- ◯ Ja
- ◯ Nein

Arbeiten Sie derzeit im Home-Office? *

❗ Bitte wählen Sie eine der folgenden Antworten:
Bitte wählen Sie nur eine der folgenden Antworten aus:

- ◯ Ja
- ◯ Nein
- ◯ teils, teils

Wie sehr treffen diese Aussagen auf Sie zu? *

Beantworten Sie diese Frage nur, wenn folgende Bedingungen erfüllt sind:
Antwort war NICHT 'Nein' bei Frage '35 [COR20J04]' (Arbeiten Sie derzeit im Home-Office?)

Bitte wählen Sie die zutreffende Antwort für jeden Punkt aus:

	**trifft nicht zu**	**trifft eher nicht zu**	**teils, teils**	**trifft eher zu**	**trifft zu**
**Ich bin froh darüber, im Home-Office arbeiten zu können.**	◯	◯	◯	◯	◯
**Ich habe durch die Arbeit im Home-Office mehr Freizeit.**	◯	◯	◯	◯	◯

Wieviele Kinder betreuen Sie zur Zeit? *

❗ Bitte wählen Sie eine der folgenden Antworten:
Bitte wählen Sie nur eine der folgenden Antworten aus:

- ◯ 0
- ◯ 1
- ◯ 2
- ◯ 3
- ◯ 4
- ◯ 5 und mehr

Wann betreuen Sie Kinder? *

Beantworten Sie diese Frage nur, wenn folgende Bedingungen erfüllt sind:
Antwort war '5 und mehr' *oder* '2' *oder* '3' *oder* '4' *oder* '1' bei Frage '37 [COR20J06]' (Wieviele Kinder betreuen Sie zur Zeit?)

❗ Bitte wählen Sie eine der folgenden Antworten:
Bitte wählen Sie nur eine der folgenden Antworten aus:

- ◯ vormittags
- ◯ nachmittags
- ◯ abends
- ◯ ganztätig

Bleiben Sie aufgrund der Covid-19-Krise, abgesehen von notwendigen Erledigungen, hauptsächlich zu Hause? *

❗ Bitte wählen Sie eine der folgenden Antworten:
Bitte wählen Sie nur eine der folgenden Antworten aus:

- ◯ Ja
- ◯ Nein

Die Umfragung ist nun beendet. Vielen Dank für Ihre Teilnahme!

Für Anregung und Kommentare nutzen Sie bitte das unten stehende Feld.

Bitte geben Sie Ihre Antwort hier ein:

Übermittlung Ihres ausgefüllten Fragebogens:
Vielen Dank für die Beantwortung des Fragebogens.
